# Preclinical Evaluation of Invariant Natural Killer T Cells in the 5T33 Multiple Myeloma Model

**DOI:** 10.1371/journal.pone.0065075

**Published:** 2013-05-31

**Authors:** Haneen Nur, Karel Fostier, Sandrine Aspeslagh, Wim Renmans, Elisabeth Bertrand, Xavier Leleu, Mérédis Favreau, Karine Breckpot, Rik Schots, Marc De Waele, Els Van Valckenborgh, Elke De Bruyne, Thierry Facon, Dirk Elewaut, Karin Vanderkerken, Eline Menu

**Affiliations:** 1 Department of Hematology and Immunology, Myeloma Center Brussels, Vrije Universiteit Brussel (VUB), Brussels, Belgium; 2 Department of Biology, Faculty of Science and Technology, Hebron University, Hebron, Palestine; 3 Department of Clinical Hematology, Universitair Ziekenhuis Brussel, Brussels, Belgium; 4 Laboratory for Molecular Immunology and Inflammation, Department of Rheumatology, Ghent University, Ghent, Belgium; 5 Department of Laboratory Hematology, Universitair Ziekenhuis Brussel, Brussels, Belgium; 6 Service des maladies du sang, Hôpital Huriez, CHRU, Lille, France; 7 Laboratory of Molecular and Cellular Therapy, Vrije Universiteit Brussel (VUB), Brussels, Belgium; University of Oslo, Norway

## Abstract

Immunomodulators have been used in recent years to reactivate host anti-tumor immunity in several hematological malignancies. This report describes the effect of activating natural killer T (NKT) cells by α-Galactosylceramide (α-GalCer) in the 5T33MM model of multiple myeloma (MM). NKT cells are T lymphocytes, co-expressing T and NK receptors, while invariant NKT cells (iNKTs) also express a unique semi-invariant TCR α-chain. We followed iNKT numbers during the development of the disease in both 5T33MM mice and MM patients and found that their numbers dropped dramatically at the end stage of the disease, leading to a loss of total IFN-γ secretion. We furthermore observed that α-GalCer treatment significantly increased the survival of 5T33MM diseased mice. Taken together, our data demonstrate for the first time the possibility of using a preclinical murine MM model to study the effects of α-GalCer and show promising results of α-GalCer treatment in a low tumor burden setting.

## Introduction

Natural killer T (NKT) cells are T lymphocytes that act as a functional bridge between the innate and the acquired immunity; therefore they have a crucial effect in tumor and pathogen resistance as well as autoimmunity [1], [2], [3]. They co-express conventional T cell (CD3) and NK cell (NK1.1) surface receptors, and furthermore, an important subset of NKT cells called invariant NKT cells (iNKTs) express a unique semi-invariant TCR α-chain encoded by Vα14.Jα18 in mice and Vα24.Jα18 in human [4]. These iNKTs can recognize glycolipid antigens presented by the class I-like major histocompatibility complex (MHC) molecule CD1d [5]. KRN7000 or α-Galactosylceramide (α-GalCer) is a synthetic glycolipid which was first discovered in a marine sponge [6], [7] and which can be presented by antigen presenting cells (APCs) such as professional dendritic cells (DCs) through CD1d [8], [9]. In this way DCs can induce activation of iNKTs, leading to an anti-tumor Th1 (IFN-γ) response or an immunosuppressive Th2 (IL-4) response [10], [11], [12]. Multiple myeloma (MM) is a B-cell malignancy hallmarked by uncontrolled accumulation of terminally differentiated monoclonal plasma cells in the bone marrow (BM) [12] which remains mostly incurable despite all the currently available therapeutic strategies. Many preliminary studies have shown critical roles of iNKTs in immune responses against a variety of carcinogen-induced and genetic tumor models when stimulated with α-GalCer [5], [13], [14], [15]. Other studies however indicated the development of iNKT anergy after α-GalCer injection, possibly due to presentation by non-professional APCs which lack the proper co-stimulatory signals [4], [14]. To overcome this problem, α-GalCer was loaded onto mature DCs which resulted in a large expansion of iNKTs, leading to a more prolonged response and induced more potent resistance to tumor development [5], [14], [16]. Clinical studies in MM patients and other patients with advanced cancer showed low biological response (low IFN-γ secretion) of iNKTs which was correlated to a dysfunction of the cells [5], [8], [16], [17], [18]. It was namely found that NKTs from cancer patients such as MM, isolated after in vivo expansion, secreted less IFN-γ after culture with 100 ng/mL α-GalCer compared to NKTs from healthy donors [8]. Similarly, Dhodapkar et al. demonstrated that in progressive MM, iNKTs are still detectable in the blood and tumor microenvironment but they have a profound deficiency in their IFN-γ production while in MGUS (monoclonal gammopathy of unknown significance) patients, the deficiency was potentially reversible [16]. This defect could be overcome in vitro as described above by using DCs loaded with α-GalCer [14], [16]. Importantly, when MM patients were injected with α-GalCer loaded DCs their circulating iNKT pool expanded 100 fold and lasted for several months, which was detectable by cell surface staining for TCR and α-GalCer-CD1d dimer [8]. It was found that iNKTs can also mediate anti-myeloma effects by cytotoxic lysis of MM cells and by activation of NK cells and other antitumor T cells such as CD8+ T cells [1], [8], [12]. This makes iNKTs interesting effectors against MM cells not only by playing a role in controlling the malignant growth of MM but also as a useful predictor of clinical outcome in MM patients [1]. However, the development of effective iNKT-cell-based immunotherapy is still a challenge and needs more investigation [5]. Furthermore, the data on NKT activity in MM patients is limited and the use of α-GalCer as a drug has not been preclinically evaluated yet in MM. Therefore, in this study, we investigated the activity and the characteristics of iNKTs in the syngeneic preclinical 5T33MM murine model, an immunocompetent model which mimics the human disease closely. Findings in this model could be translated into a clinical setting and thereby contribute to the quick development of new therapeutic strategies [19].

## Materials and Methods

### Patients

Patient samples have been collected with the approval of the Ethics Board of UZ Brussel (BUN143201215265) and the Tumourbank of Lille (CSTMT102). Samples are considered “waste samples” and therefore under Belgian legislation, informed consent is not required. In short it is stated that in case of waste samples for scientific research, the consent is assumed to be given unless the patient specifically indicates otherwise. Patients at our institution are aware of this right to refuse the use of the samples. All patient samples were de-identified and analyzed anonymously. Each patient received a number and we only had access to the number.

### 5T33MM Mouse Model

C57Bl/KaLwRij mice were purchased from Harlan CPB (Horst, the Netherlands). The mice were 6 to 8 weeks old when used. They were housed and treated following the conditions approved by the Ethical committee for animal experiments, VUB (license no. LA1230281). Approval for these specific experiments was obtained by the committee with approval number 09-281-5. The animal ethics meet the standards required by the UKCCCR Guidelines (UKCCCR, 1998). The 5T33MMvv model originated spontaneously in C57BL/KaLwRij mice as described previously [19], [20] and have since been propagated by intravenous injection of the diseased BM into young syngeneic recipients [21], which in turn develop myeloma in 3-4 weeks. In short, mice were sacrificed when showing signs of morbidity and the BM was flushed out of the femurs and tibiae and crushed out of the vertebrae. The BM cells were suspended in serum free medium (RPMI 1640 (Lonza, Belgium), supplemented with penicillin-streptomycin, glutamine, and MEM NEAA-pyruvate (Lonza)). The cells were then purified by Lympholyte M (Cedarlane, Hornby, Canada) gradient centrifugation at 1000 g for 20 min. The cell band on top of the gradient contained enriched 5T33MM cells, with a purity reaching 85%, as measured by flow cytometric analysis. Viability was more than 95%. The model resembles the human disease closely, with infiltration in the BM and secretion of paraprotein. As the spleen is a hematopoietic organ in mice, infiltration also occurs in spleen [22]. There is furthermore to a lesser extent tumor growth in the extravascular compartment of the liver.

### Flow Cytometry

For detection of murine iNKTs, cells were washed and resuspended in PBS containing 1% BSA and 0.02% sodium azide. Cells were incubated for 5 min at 4°C with FCR blocking reagent (Miltenyi Biotic, Bergisch Gladbach, Germany), followed by addition of anti-α-GalCer/CD1d tetramer-APC (Proimmune, Oxford, UK), and anti-TCR-β-PE (clone H57-597, BD biosciences, Erembodegem, Belgium) for 30 min at 4°C. 7-AAD (BD biosciences) was added in the last 15 min, followed by washing and resuspension in FACS buffer.

CD1d expression on 5T33MM+and – cells was assessed by triple staining with anti-CD1d-PE (clone 1B1, BD biosciences), anti-CD11b-FITC (clone M1/70, BD biosciences) and anti-5T33MM idiotype [22] for 30 min at 4°C. For 5T33MM, anti- IgG1-APC (clone X56, BD biosciences) was used as a second step. 5T33MM cells are considered 5T33MM+ and CD11b-. Appropriate isotype controls were used. All samples were acquired on a FACS Canto and analysed with FACS Diva software (BD biosciences).

For human iNKT analyses, an antibody mixture composed of anti-CD3-FITC (clone sk7, BD biosciences)/anti-CD16CD56-PE (clone 3G8, My31, Analis, Ghent, Belgium)/anti-CD45-Texas Red (clone J33, Analis)/anti-CD19- PC5 (clone J3-119, Analis) was used to determine the amount of T lymphocytes on peripheral blood cells. In a second tube, anti-CD3-PC5 (clone ucht1, Analis), anti-TCRVα24-FITC (clone 6B11, BD biosciences) and anti-TCRVβ11-PE (clone C21, Analis,) was used to gate iNKT cells.

### Liver Perfusion and MACs-sorting

Mice were anesthetized i.p. with a 75 mg/kg ketamine (CEVA Santé Animale, Libourne, France) –50 mg/kg medetomidine hydrochloride (Virbac, Burgdorf, Germany) mixture and the abdomen was opened immediately, renal veins were tied and the inferior vena cava and portal veins were cannulated simultaneously with 18G needles. Livers were perfused with 25 mL of 10 mM EDTA/PBS (pH 7.4) at 7 mL/min within a closed sterilised catheter system using a peristaltic pump (Gilson, France). The flow-out was collected, washed and lysed with 2 mL Red Blood Cell lysis buffer for 1 minute, and neutralized with RPMI-1640 medium (Lonza), supplemented with 1% penicillin/streptomycin and 10% FCS (Biochrome AG, Berlin, Germany) [23]. Purity of the liver iNKT cells was determined by flow cytometry and mentioned in the results.

Spleen iNKT cells were crushed out of the spleen and purified through MACS sorting using α-Galcer/CD1d tetramer-APC and anti-APC microbeads (Miltenyi Biotec,) as previously described [24]. Briefly, cells were labelled with the tetramer for 30 min on ice and then the microbeads were added for 15 min. Cells were passed over an LS column and the purity of the splenic iNKT cells in the positive selection was determined by flow cytometry and mentioned in the results.

### Generation of Dendritic Cells and Co-culture with iNKTs

Murine BM progenitor cells were isolated by flushing the content of the femur and tibia of naive mice with sterile PBS as described previously [25]. DCs were cultured from these BM progenitor cells and plated in 100×20 mm cell culture dishes (Greiner Bio-One, GmbH, Germany) at 1×10^6^/2 mL in supplemented RPMI-1640 media with 5% FetalClone I (FCI, Hyclone, Logan, USA). 20 ng/mL of recombinant mouse granulocyte macrophage colony stimulating factor (GM-CSF, R&D systems, Oxon, UK) and 50 µM β-mercaptoethanol (β-ME, Sigma-Aldrich, Belgium) were added to the culture at day 0. On day 3, 50% of the volume fresh medium with GM-CSF and β-ME was added. On day 6, 50% of the media were completely refreshed. On day 7, the immature DCs were harvested and lipopolysaccharide (LPS, 100 ng/mL, Sigma-Aldrich) was used to mature DCs overnight in the presence of 100 ng/mL alpha-galactosylceramide (α-GalCer) or vehicle (0.5% Tween-20 in PBS). α-GalCer, dissolved in DMSO at 1 mg/mL was kindly provided by Dr. S Van Calenbergh (University of Ghent, Belgium) and kept at −20°C. On day 8, after extensive washing, α-GalCer loaded/unloaded DCs (2×10^5^/well) were co-cultured with iNKTs (5×10^4^/well) in supplemented RPMI-1640 medium with 10% FCS at 4∶1 ratio and incubated for 72h at 37°C and 5% CO_2_.

### Cytokine Levels

Supernatant from in vitro co-cultures was collected to determine mouse IFN-γ (eBioscience, Vienna, Austria) and IL-4 (R&D systems, Oxon, UK) concentrations by ELISA according to the manufacturer’s instructions. Cytokine level was also determined in serum collected from mice during the time course of the disease after intraperitoneal (i.p.) administration of 2 µg α-GalCer.

### Real-time Quantitative PCR (RT-PCR)

Total RNA from 3×10^6^ NKT cells was extracted by using the RNeasy kit (Qiagen, the Nederlands). Total RNA was reverse transcribed using the Verso cDNA synthesis kit (Thermo Scientific, CO, USA), according to the manufacturer’s instructions. RT-PCR was performed using Maxima® SYBR Green/ROX qPCR Master Mix (Fermentas, Germany) on an ABI Prism 7900 Fast instrument using gene-specific primers. The primer sequences for Vα14 were as follows: forward primer: 5′- AGG TAT GAC AAT CAG CTG AGT CCC -3′ and reverse primer: 5′- CTA AGC ACA GCA CGC TGC ACA -3′. The PCR cycles consisted of an initial denaturation step at 50°C for 2 min and 95°C for 10 min followed by 40 cycles at 95°C for 15 s and 60°C for 1 min. Each sample was amplified in triplicate. The relative standard curve method was used to quantify the relative Vα14 expression in the 5T33MM cells and abl was the reference gene. Relative standard curves (25, 5, 1, 0.2 ng) were prepared using cDNA from 5T33MM control samples.

### Statistics

Mann-Whitney and student’s t test were used for statistical analyses and Kaplan-Meier analysis was used for the survival assay. *p<0.05* was considered significant.

## Results

### The Evolvment of iNKT Numbers in MM

**Figure 1:**
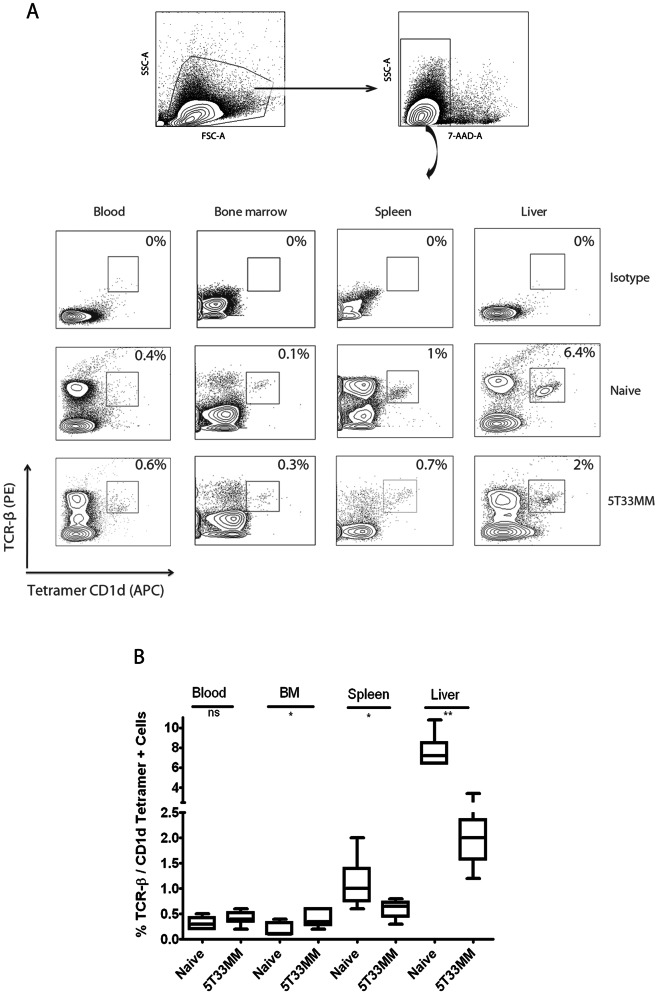
iNKT numbers in the 5T33MM model. (**A**) Representative FACS analysis of murine iNKT cells from blood, BM, spleen and liver in naive and 5T33MM mice. Live cells (7-AAD negative) were stained with α-GalCer/CD1d tetramer which specifically binds to Vα14 of the invariant TCR and with TCR-β. Double positive iNKT cells were gated. The percentages are indicated in each plot. (**B**) Box plots of the distribution of iNKT number data, obtained from 6 mice from independent experiments. Differences between naive and 5T33MM cells in BM, liver and spleen are significant (* and ** indicate p<0.05 and p<0.005, Mann-Whitney test).

**Figure 2:**
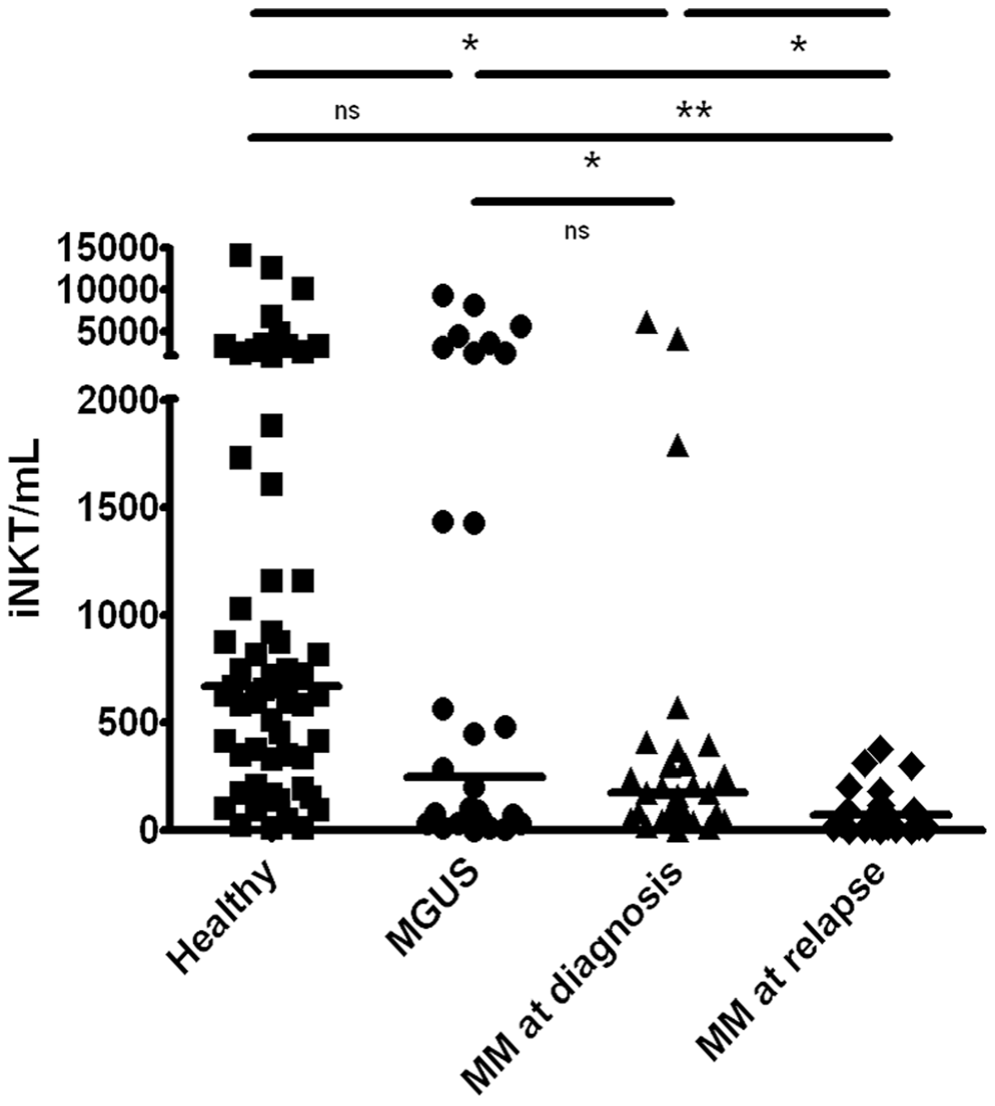
iNKT numbers in MM patients. Human iNKT (number/mL) were analyzed in blood samples of 51 Patients (mean age is 64 years old) and 62 healthy donors (mean age is 66 years old) by flow cytometry. Total number of iNKT cells were calculated by determining the % iNKT cells (CD3, TCRVα24 and TCRVβ11+ cells) on total T lymphocyte number (* and ** indicate p<0.05 and p<0.01, Student t-test, ns =  not significant).

**Figure 3:**
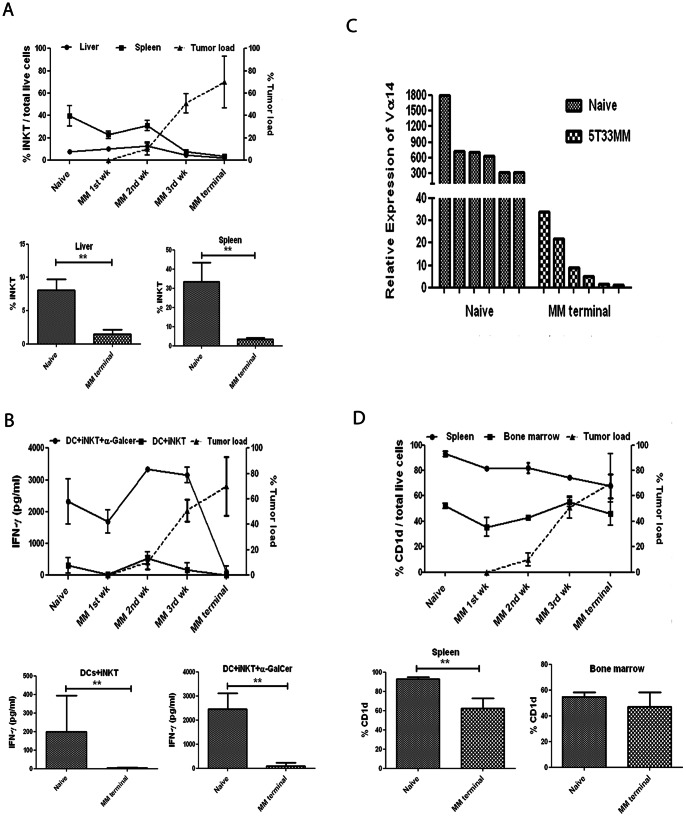
iNKT frequency and activity during the development of the disease in the 5T33MM model. **(A, upper panel)** Frequency of iNKTs in liver and spleen during the course of the disease, analyzed by flow cytometry as was done in figure 1A. Mice were isolated at different time points and liver and MACS-sorted splenic iNKTs were stained with α-GalCer/CD1d tetramer. Plasmacytosis (tumor load) was assessed on May-Grünwald Giemsa stained cytosmears of the isolated BM. The mean ± SD of 3 mice in independent experiment is shown. **(A, lower panel)** The mean % iNKT cells in liver and spleen of naive and end stage 5T33 mice (n = 6, ** indicates p<0.01, Mann-Whitney test). **(B, upper panel)** In vitro iNKT activity. Mice were isolated at different time points and liver iNKTs were co-cultured with α-GalCer loaded or unloaded DCs for 72 h. IFN-γ secretion in the co-culture was measured by ELISA. The mean ± SD of 3 mice in independent experiment is shown. **(B, lower panel)** The mean IFN-γ secretion of DC co-cultures with liver iNKT cells of naïve and end stage 5T33 mice (n = 6, ** indicates p<0.01, Mann-Whitney test). (**C**) Gene expression of the Vα14 receptor of liver iNKTs of healthy and diseased 5T33MM was assessed by RT-PCR (n = 6). **(D, upper panel)** Expression of CD1d in live spleen and BM cells in the 5T33MM model during the course of the MM disease compared to tumor load. Mice were isolated at different time points of the disease and spleen and BM cells were double stained for CD1d and 5T33MM. The mean ± SD of 3 mice in independent experiment is shown. **(D, lower panel)** The mean CD1d expression in spleen and BM cells of naive and end stage 5T33 mice (n = 6, ** indicates p<0.01, Mann-Whitney test).

We first investigated the frequency of iNKTs in the blood, BM, spleen and liver of both healthy and terminally diseased 5T33MM mice by co-staining the cells with anti-TCR-β and CD1d tetramer. A representative staining is shown in Figure 1A. Taking the average of 6 mice (figure 1B), the highest percentage of iNKTs was found in the liver (naive 7.6%±1.64) with a significant decrease in 5T33MM mice (2.1%±0.73). The percentage was also significantly decreased in spleen from 1.1%±0.48 in naive to 0.6%±0.18 in 5T33MM mice, while in BM we saw a significant upregulation of iNKT number from 0.18%±0.13 to 0.43%±0.16 and no significant differences in the blood. The upregulation in the BM could suggest tumor surveillance by the iNKTs. Human iNKT number was also determined in blood samples of 55 MM patients (29 at diagnosis and 26 at relapse) and 28 patients with Monoclonal Gammopathy of Unknown Significance (MGUS). We saw a median of 670 iNKT/mL in the 62 healthy donors and this number gradually decreased to 248, and 178 in MGUS and newly diagnosed patients. There was a significant decrease to 74 iNKT/mL in relapsed patients (Figure 2). As we wished to determine the moment when iNKT numbers dropped, we followed the frequency of iNKTs in the liver and spleen of MM mice during the development of the disease. We analyzed the number of iNKTs in the first, second, third and terminal week of the disease. Liver [23] and MACS-enriched spleen [24] cells were stained with α-GalCer/CD1d tetramer (Figure 3A, upper panel). We found 40%±9.15 MACS-enriched spleen cells in healthy mice and this number gradually dropped to 8%±1.2 at the third week of disease. This coincided with a sharp increase in tumor load from 10%±5.29 at week 2 to 51%±8.71 at week 3, suggesting a correlation. Similar results were found with the liver iNKT cells which dropped from 7.6%±1.5 to 4.7%±0.47 at week 3. There was a significant decrease in both liver and spleen iNKT number when comparing naive and end stage diseased animals (Figure 3A, lower panel, n = 6). These findings relate to what we saw in patients, namely a sharp decline in iNKT number at the advanced/relapsed stage of MM disease.

### IFN-γ Secretion in MM

To analyze the activity of iNKTs in vitro and to determine if lower iNKT number results in lower cytokine secretion, total liver iNKTs from different time points of the disease (Figure 3B, upper panel) were co-cultured with naive matured bone marrow-derived DCs in the presence or absence of 100 ng/mL α-GalCer. Liver iNKTs are more active compared to spleen iNKTs (data not shown) [1], [4]. Naive iNKTs could secrete up to 2.3±0.71 ng/mL IFN-γ when stimulated with α-GalCer, and this level increased with the progression of MM to reach 3.3±0.017 ng/mL at week 2. However, the IFN-γ secretion of the iNKTs dropped to undetectable levels upon further progression of the disease (week 4). This drop was significant (Figure 3B, lower panel, n = 6). Similar results were obtained using DCs derived from BM of 5T33MM mice, implying no defect in the DCs (data not shown). Unstimulated iNKTs secreted a background level of up to 300±251 pg/mL IFN-γ during the course of the disease. These data indicate that a drop in cytokine secretion from the iNKTs could be preceded by a decline in iNKT number. To confirm that this is an actual decline in iNKT number and not the result of internalization of the Vα14 receptors, we performed RT-PCR of the Vα14 receptor of healthy and terminally diseased murine liver iNKTs (Figure 3C). If the receptor would be internalized, mRNA levels would still be high. One of the 5T33MM samples was used as reference and the other values were calculated relative to this. We could find that the mRNA expression of Vα14 correlated to the number of iNKTs measured by FACS with a clear drop in iNKT number in terminally diseased 5T33MM mice. Only very slight IL-4 production (6±2 pg/mL) was observed when α-GalCer was administered to the culture (data not shown) indicating that liver iNKTs are skewed to a Th1 profile and can therefore be used as an immunotherapeutic tool in MM when activated by adjuvants such as α-GalCer.

### CD1d Expression

It has been described previously that the expression of CD1d molecules is significantly downregulated in patients with advanced stages of MM compared to healthy controls, MGUS and early myeloma patients, suggesting a reason for loss of iNKT activity [26]. To investigate if this is similar in the 5T33MM model, we followed the expression of the CD1d molecules on total spleen and BM cells during the course of the disease (Figure 3D, upper panel). Results showed a small downregulation of CD1d expression on spleen cells starting from week 2. Approximately, 93%±2.1 CD1d expression was seen in naive spleen cells while the level decreased with the progression of the disease to 82%±3.9 in week 2, 74%±1.6 in week 3 and 68%±9.3 at end stage. The reduction at end stage was significant (Figure 3D, lower panel, n = 6) In naive mice, CD1d was less expressed on BM cells compared to spleen cells (52%±2.1) and in tumor bearing mice, the expression declined in week 1 and week 2 (35%±7.2 and 43%±1.6 respectively) but did not decline further at the terminal point. Furthermore we analyzed CD1d expression on the MM cells themselves and found that the receptor was highly expressed (79%±6.2 in spleen and 67.7%±2.3 in BM) in week 1 and that this did not alter during the course of the disease (82.8%±4.1 in spleen and 75%±7.5 in BM) at end stage (data not shown).

### In vivo Response to α-GalCer

**Figure 4:**
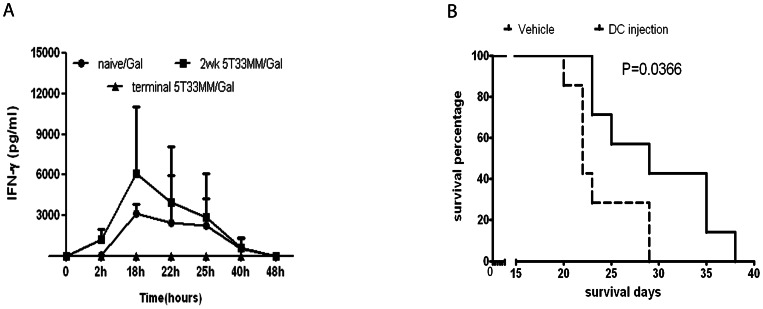
iNKT activity in vivo. (**A**) In vivo cytokine secretion after i.p. administration of 2 µg α-GalCer in 200 µl of PBS to healthy, non-terminal and terminal sick 5T33MM mice. Serum was collected during a time course, responses were followed by determining IFN-γ and IL-4 levels by ELISA (mean ± SD is shown from 5 mice in each group). (**B**) Kaplan-Meier survival assay. Mice were intravenously injected with a single dose of matured DCs (6×10^5^ cell/mouse) loaded with α-GalCer (100 ng/mL) or unloaded at the same day of inoculation with 5T33MM cells (n = 8 mice/group, p = 0.0366). Vehicle mice had an average survival of 22 days, α-GalCer treated mice 29 days.

As we wished to investigate whether the kinetics of the in vivo response of iNKTs to α-GalCer altered during the course of the disease, we injected naive, non-terminal and terminal diseased 5T33MM mice with α-GalCer. Cytokine secretion in the peripheral blood was followed at different time points after injection (Figure 4A). It has been described that an IL-4 peak appears faster than an IFN-γ peak [5], [14]. In the 5T33MM model, the serum level of IFN-γ peaked at 18h in naive and non-terminal diseased mice and returned to baseline by 48h, however, the response of IFN-γ in diseased mice was twice (6000±4925 pg/mL) that measured in naive mice, confirming the possibility of inducing Th1 responses with α-GalCer in vivo in healthy and diseased mice. These findings are consistent to previous results obtained in different mouse tumor models [14], [27]. No response could however be detected from terminally diseased mice. IL-4 was detected only in naive mice after 2h of α-GalCer administration (600±489 pg/mL) and completely disappeared at 18h (data not shown). We finally evaluated the effect of α-GalCer on the survival of MM mice. When mice were injected i.p. with α-GalCer on the same day of 5T33MM inoculation, we saw no significant survival increase compared to vehicle. Vehicle mice had an average survival of 30 days while α-GalCer injected mice 32 days (data not shown). However, the survival was significantly increased when mice were injected with α-GalCer loaded DCs on the same day of 5T33MM inoculation (29 days survival) compared to mice injected with unloaded DCs (22 days survival, Figure 4B), indicating that DC presentation of α-GalCer is necessary to have an anti-tumoral effect.

## Discussion

Natural killer T (NKT) cells can be activated by the synthetic glycolipid α-GalCer and this has been shown to lead to anti-tumor responses in different mouse models such as the transgenic Eµ-myc B cell lymphoma and AML-ETO9a leukemia mouse model [28]. In MM however, not much is known about the preclinical possibilities of harnessing NKT cells due to the lack of appropriate mouse models. In this article we demonstrate the ability to use the immunocompetent 5TMM model as a murine model to study the effects of α-GalCer on iNKTs. First we wished to analyze the presence of iNKTs in healthy and MM diseased mice and to determine whether this correlated to patient data. We found a significant drop in liver and spleen iNKT number, while we also saw a small but significant upregulation of iNKTs in the BM, indicating that a part of the iNKTs migrated to the tumor. The general reduction in iNKTs correlated to a reduction in circulating iNKT we observed when comparing healthy donors to MM patients with relapsed disease. We furthermore saw that this decrease was gradual, with already a reduction in MGUS patients, a further reduction in newly diagnosed MM patients but with a most pronounced decline in patients with relapsed disease. This has also been found by others who described that in MM patients, the progression of the disease is associated with loss of IFN-γ secreting iNKTs [16], [28]. We next wished to determine when the drop in iNKT number actually occurred in the 5T33MM model so we performed time course experiments. We found that there was a sharp decrease at week 3 of the disease which coincided with a sharp increase of tumor load. Loss of iNKT cells was furthermore confirmed by RT-PCR for the Vα14 receptor. This is the invariant chain of the TCR receptor, associated with iNKTs. A reduction of this receptor in the total cell population indicates a reduction in NKT cell number. We next investigated whether this decline in iNKT number lead to a decrease in total IFN-γ secretion in vitro. When we co-cultured iNKTs of different disease stages with α-GalCer loaded DCs, we saw indeed a reduction in IFN-γ secretion at week 4. Interestingly, at week 3, IFN-γ expression is still high, indicating that the loss of IFN-γ production comes later than the drop in iNKT numbers. A possible reason for loss of iNKT activity in cancer patients has been given by van der Vliet et al [29] who found in melanoma and renal cell cancer patients that the circulating myeloid dendritic cells have an altered cytokine profile which leads to a loss of Th1 activity in the remaining iNKT cells. We compared the possibility of BM derived DCs from healthy and diseased mice to activate iNKTs, but found no difference. It has also been previously described [26] that loss of CD1d, the lipid presenting molecule, could be the reason for the loss in iNKT activity, therefore we followed its expression during the course of the disease, both on total spleen and BM cells, containing the APCs and also on the 5T33MM cells as it has been described that MM cells can function as APCs themselves [28]. We saw a small but significant decline in CD1d expression in spleen cells but not in BM cells, indicating that this is not the major reason for loss of IFN-γ secretion. 5T33MM cells expressed high levels of CD1d that did not alter during the course of the disease. This is in contrast with Spanoudakis et al [26] who found that CD1d was significantly downregulated in advanced stages of MM and myeloma cell lines. We performed co-culture experiments in which 5T33MM cells were used as APCs to trigger cytokine secretion by healthy iNKTs but we found no activation (data not shown). This is in contrast to a study which demonstrated the possibility of injecting irradiated tumor cells loaded with α-GalCer in the Vk*myc MM model which led to a reduction in tumor load if injected before inoculation with tumor cells [28]. The 5T33MM cells seem to be missing the necessary co-stimulatory molecules such as CD40, CD70, CD80, CD86 and OX40L [11], [30], [31], [32], as they only expressed 5% CD40, 8% CD80 and 2% CD86 on their membrane (data not shown) in contrast to normal APCs to elicit a response. Another possibility is that the 5T33MM cells display a non functional form of CD1d, missing the full cytoplasmic tail which is necessary for activity [33]. Further investigation is necessary to examine this CD1d deficiency. We finally examined the possibility of inducing an anti-tumor response in vivo by injecting α-GalCer. It has been shown in vivo that inducing a Th1 response in iNKTs leads to the recruitment of other immune cells against the tumor [34]. We first looked at the in vivo response of iNKTs to α-GalCer by measuring IFN-γ secretion in the peripheral blood of the mice. Treating week 2 diseased mice resulted in a robust cytokine response in vivo; similar to that of naive mice, but when treating end-stage diseased mice this activity was lost, confirming the cytokine loss we saw in vitro. As it has been shown that α-GalCer can fail to elicit a clear Th1 or Th2 response in iNKTs, several analogues of α-GalCer have been discovered and modified to improve either the Th1 or Th2 response [35]. OCH has been shown to give a stronger Th2 response, suitable for auto-immune diseases [11], [36]; both the carbon glycosidic analogue (α-C-GalCer) and naphthylurea 6″- derived α-GalCer (NU- α-GalCer) have been shown to be superior Th1 polarizers in B16 melanoma cells [37], [38], [39]. The group of Tyznik demonstrated that plakoside-based antigens were also able to stimulate mouse and human iNKTs and cause a prolonged systemic synthesis of IFN-γ. [40]. Studies of these new derivatives in MM are however lacking and should be further explored. We finally performed a Kaplan-Meyer survival analysis in which we treated 5T33MM inoculated mice with either vehicle loaded DCs or α-GalCer loaded DCs. We found that α-GalCer treatment significantly prolonged mouse survival from 22 days to 29 days. This opens the road to possibilities of combination therapy in which the tumor is targeted with a known cytotoxic drug and the residual disease is targeted by iNKT activation. Recently, a clinical trial using α-GalCer loaded DCs combined with the immunomodulatory drug Lenalidomide led not only to the activation of NKT cells but also to the activation of the other downstream components of the innate immunity including NK cells, monocytes and eosinophils which can collectively induce tumor regression in MM. [41].

Taken together, our data demonstrate for the first time the possibility of using a murine model as a preclinical MM model to study the effects of α-GalCer on iNKTs. This study also opens up the possibility of using the 5T33MM model to evaluate other immunotherapeutics in MM such as DC vaccinations. As our data show that there is an iNKT impairment at the end stage of the disease, future studies should focus on overcoming this impairment by combining α-GalCer or its newly Th1 polarizing analogues [27] with immunomodulatory agents. Combination studies with known cytotoxic drugs could give novel insights into how the MM tumor can be targeted by multiple pathways, leading ultimately to improved treatment of MM patients.
